# Radiological presentation of chondromyxoid fibroma in the sellar region: A CARE-compliant article and literature review

**DOI:** 10.1097/MD.0000000000009049

**Published:** 2017-12-08

**Authors:** Shuai Shen, Miao Chen, Rachel Jug, Cheng-Qian Yu, Wan-Lin Zhang, Lian-He Yang, Liang Wang, Juan-Han Yu, Xu-Yong Lin, Hong-Tao Xu, Shuang Ma

**Affiliations:** a Department of Neurology, Shengjing Hospital of China Medical University, Shenyang, Liao Ning, China; b Department of Pathology, Duke University Medical Center, Durham, NC; c Department of Pathology, First Affiliated Hospital of China Medical University and College of Basic Medical Sciences, Shenyang, Liao Ning, China.

**Keywords:** chondromyxoid fibroma, computed tomography, magnetic resonance imaging, sellar region, skull neoplasm

## Abstract

**Rationale:**

Chondromyxoid fibroma (CMF) is a rare benign bone neoplasm which often occurs in the lower extremities. Little is known about the radiological and histological presentation of CMF in the sellar region.

**Patient concerns:**

A 16-year-old Asian male presented to the hospital 12 months ago with bilateral diplopia involving right visual fields, intermittent headaches, and dizziness.

**Interventions:**

After the patient underwent enough examinations, the lesion was surgically removed by curettage.

**Diagnosis:**

Postoperatively, the lesion was pathologically confirmed to be CMF.

**Outcomes:**

There was no recurrence at the 12-month follow-up.

**Lessons:**

To the best of our knowledge, this is the second reported case of CMF in the sellar region which was clinically suspected to be a pituitary macroadenoma, craniopharyngioma, or schwannoma due to its location and radiographic features. We reviewed the morbidity, symptoms, radiographic features, pathological findings, and differential diagnosis of CMF. Because of its rarity, attention should be paid to avoid misdiagnosis of this lesion.

## 1 Introduction

Chondromyxoid fibromas (CMF) are extremely rare benign cartilaginous neoplasms that account for <1% of all bone tumors.^[1]^ It was first described by Jaffe and Lichtenstein^[2]^ in 1948. Most CMFs are located in the metaphyseal region of long bones; the knee is the most common site where CMF occurs (40%) where it may extend to the epiphyseal line and even rarely about the articular surface. CMF of the skull base is rather rare. There are a few reports of CMF at the skull base including ethmoid bone, frontal bone, temporal bone, occipital bone, and sphenoid bone.^[3]^ To the best of our knowledge, there is only 1 case published in the English literature of CMF involving the sellar region. The majority of cases occur in the second and third decades of life, with approximately 75% of cases occurring before the age of 30 years. There is no significant sex predilection.^[4–7]^ The characteristic radiologic appearance of CMF is a well-circumscribed, lytic defect with scalloped, sclerotic margins similar to a metaphyseal fibrous defect. Histologically, CMF is characterized by lobules of spindle-shaped or stellate cells surrounded by abundant myxoid or chondroid intercellular matrix with a varying number of differently sized multinucleated giant cells.^[8]^ Because this is an infrequent site of involvement, CMF in the skull base can be easily misdiagnosed by histology as chondrosarcoma or chordoma, or radiologically as craniopharyngioma or pituitary macroadenoma, because these neoplasms share some similarities.^[9]^ Herein, we report a rare case of CMF in the sellar region. The lesion as appeared radiologically similar to craniopharyngioma, pituitary macroadenoma, and schwannoma, however, was confirmed histologically after surgical resection to be CMF.

## 2 Case presentation

A 16-year-old Asian male presented to the hospital 12 months ago with bilateral diplopia involving right visual fields, intermittent headaches, and dizziness. His visit to the hospital was prompted by worsening of symptoms over the preceding 2 weeks. No other neurological deficits were identified. The patient had no history of trauma or cranial nerve abnormalities.

### 2.1 Materials and methods

The patient underwent computed tomography (CT) and magnetic resonance imaging (MRI) examinations. Pathology examination were performed, the resected tissues were embedded in paraffin blocks, and sectioned. Immunohistochemistry was performed using an SP kit (Maixin Biotechnology, Fuzhou, Fujian, China) according to the manufacturer's instructions. The sections were incubated 2 hours at 37°C with the following primary antibodies: S100 (1:200, Dako, Carpinteria, CA) and glial fibrillary acid protein (GFAP) (1:200, Dako). This study was prospectively performed and approved by the institutional Ethics Committees of China Medical University and conducted in accordance with the ethical guidelines of the Declaration of Helsinki. Written informed consent was obtained from the patient for the publication and accompanying images.

## 3 Results

**Figure 1:**
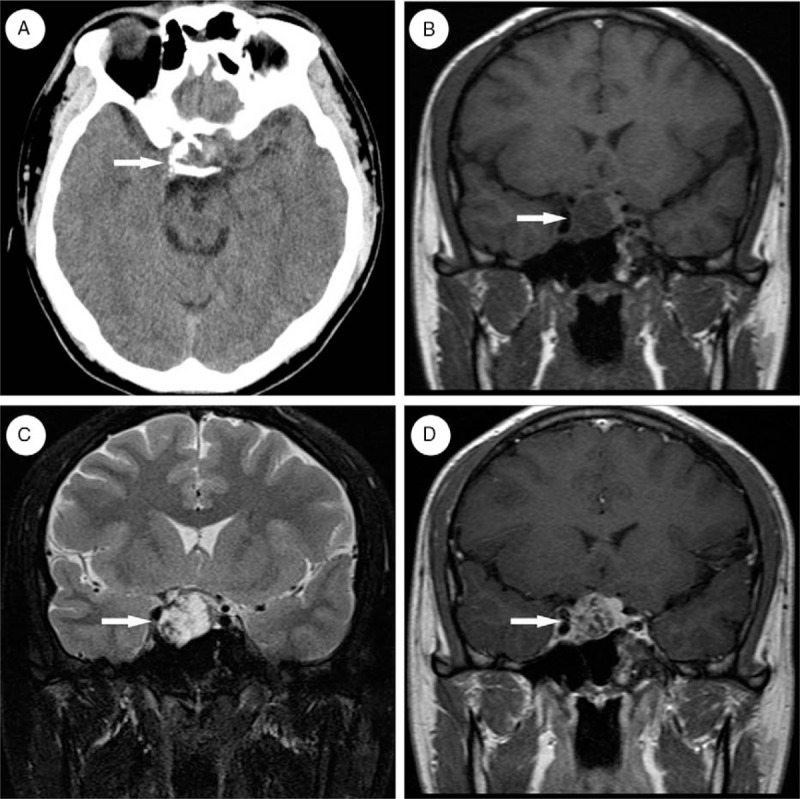
(A) Computed tomography (CT) scan showed intermediate-to-high density shadow of clusters in sellar region with multiple calcification nodules at the boundary, lesion was marked with white arrow. (B) Hypointensity T1-weighted image, lesion was marked with white arrow. (C) Hyperintensity T2-weighted image, lesion was marked with white arrow. (D) Inhomogeneous contrast enhancement of T1-weighted image, lesion was marked with white arrow.

The CT scan showed a cluster of intermediate-to-high density shadows in sellar region with multiple calcified nodules at the boundary, suggesting the lesion might have been a craniopharyngioma (Fig. 1A). A MRI of the sellar region demonstrated enlargement and sinking of the base of the sellar region. A T2-weighted image showed hybrid signals, whereas the T1-weighted image displayed a hypo-intense region (2.2 × 1.6 × 2.0 cm^3^) with inhomogeneous enhancement (Fig. 1B–D). The optic chiasm had been compressed upwards by the lesion. The MRI results were compatible with a differential diagnosis of pituitary macroadenoma or schwannoma.

**Figure 2:**
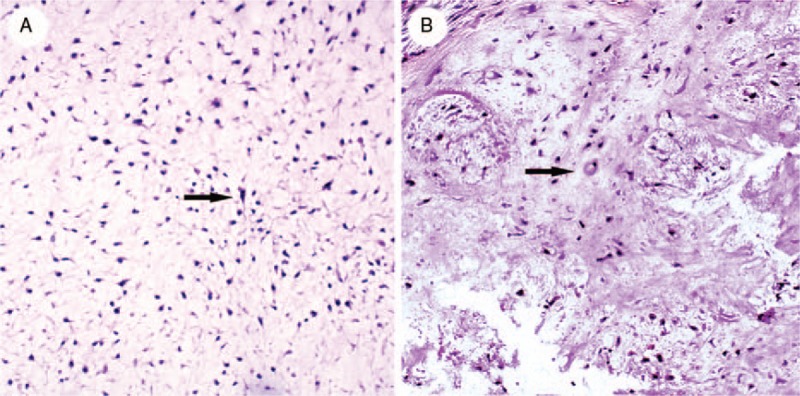
(A) Stellate or spindled-shaped cells surrounded by fibrous myxoid intercellular matrix, marked with black arrow. (B) Immature chondrocytes surrounded by fibrous myxoid intercellular matrix, marked with black arrow.

The lesion was surgically removed by curettage. Histologic examination revealed immature chondrocytes and stellate or spindle-shaped cells surrounded by a fibromyxoid intercellular matrix (Fig. 2 A, B). Immunohistochemical (IHC) staining was positive for S100 protein but negative for GFAP, supporting the diagnosis of CMF.

## 4 Discussion

Although CMF was first described by Jaffe and Lichtenstein^[2]^ back in 1948, it is a relatively rare lesion. A review of the English literature identified only 1 other published case of CMF in the sellar region. Symptoms of CMF vary depending on the location and the size of the tumor. Patients can suffer from bony swelling, dysarthria, neuralgia, diplopia, facial pain, exophthalmos, headache, polyuria, and polydipsia.^[5,6,10]^ In our case, the patient presented with diplopia, occasional headaches, and dizziness, which is consistent with the presentation seen in the previously reported case. Imaging studies are crucial for the diagnosis of CMF; CT scans of CMF commonly show an osteolytic lesion with a sclerotic margin.^[4]^ On MRI examination, T1-weighted images show a hypointense signal with inhomogeneous contrast enhancement, whereas T2-weighted images show a hyperintense signal.^[5,11]^ Hypointense T1-weighted images and hyperintense T2-weighted images indicate the presence of myxoid and cartilage tissue, respectively. Inhomogeneous contrast enhancement suggests the presence of fibrous tissue. In our case, the CT result showed a cluster of intermediate-to-high density shadows with mineralization at the margin, MRI studies revealed hypointensity on the T1-weighted image with inhomogeneous contrast enhancement and the T2-weighted image showed a hybrid signal. These features are consistent with those previously described in the other reported case of sellar CMF. However, the high-density shadow on CT and isointensity on the T2 image indicated there were also foci of calcifications present which is an uncommon feature in CMF (13%).^[12]^

Pituitary macroadenoma, craniopharyngioma, and schwannoma were on the list of differential diagnoses due to the location and radiographic features of this patient's lesion. Pituitary adenomas are the most common tumors in the sellar region, and in cases where the tumor exceeds 10 mm in size, they are defined as macroadenomas.^[13]^ Hormone-secreting pituitary adenomas cause 1 of several forms of hyperpituitarism. Symptoms associated with functional pituitary adenomas depend on the predominating cell type within the neoplasm and the hormone secreted. This phenomenon is not seen within CMF as it is not a tumor composed of pituicytes with functional hormone synthesis capacities.^[14]^ However, pituitary adenomas, especially macroadenomas, may present with visual field defects which arise from compression of the optic nerve by the tumor, which could mimic a presenting symptom of CMF involving the sellar region. The presence of absence of contrast attenuation can vary depending on the variable presence of hemorrhagic, cystic, and necrotic components. Adenomas which are solid, without hemorrhage, typically have attenuation similar to brain and demonstrate moderate contrast enhancement. MRI typically shows isointensity to gray matter in T1 and T2-weighted images. Pituitary macroadenoma is easy to differentiate from CMF radiologically in the presence of foci of calcification as it is extremely rare for a pituitary macroadenoma to demonstrate calcification. However, the majority of radiographic features of pituitary macroadenoma are otherwise nearly the same as CMF without calcifications.^[4,5,13–17]^

Craniopharyngioma commonly occurs in the sellar region of the brain, near the pituitary gland, and often involves the third ventricle, optic nerve, and pituitary gland.^[18]^ Craniopharyngiomas may cause symptoms associated with the increased pressure on the optic tract and pituitary gland, which could include obesity, delayed development, visual impairment, and swelling of the optic nerve.^[19,20]^ Craniopharyngiomas have lobulated contours as a result of usually multiple cystic lesions. The low-density shadow indicates cystic degeneration, which is not apparent in CMF. Calcification is commonly seen in craniopharyngiomas, which, while present in our case, is not a feature typically associated with CMF. MRI features of craniopharyngiomas are distinct from CMF, including an isointense to hyperintense T1 signal, and variable or partly hyperintense T2 signal.^[21,22]^

Schwannoma is a benign tumor of schwann cell origin, and most intracranial cases arise from the vestibulocochlear nerve and are termed “acoustic neuroma.”^[23,24]^ Schwannomas arising in the sellar region are very rare. Clinical presentation depends on the size of the tumor, the nerve on which it originates, and its exact location along the nerve tract and in association with cortical matter. On CT images, schwannomas are typically isodense to the brain and can be difficult to identify. MRI is the optimal imaging modality for identifying intracranial schwannomas because of superior contrast resolution and exquisite anatomical details, particularly with the high-resolution T2 sequence.^[23,24]^ CT scans of CMF reveal an intermediate-to-high density shadow (with foci of calcification) or intermediate-density shadow (without foci of calcification) which differs from the low-density shadow of schwannomas.

## 5 Conclusions

In summary, CMF in the skull base, especially in the sellar region, is very rare. Symptoms can vary depending on the adjacent structures involved. We reported a case of CMF in the sellar region which compressed the optic chiasm that was suspected to be craniopharyngioma, schawannoma, or pituitary macroadenoma based on its radiographic appearance and location. We reviewed the morbidity, symptoms, radiographic features, and differential diagnosis of CMF in sellar region, which may be helpful in clinical practice. Because of its low frequency in the skull base, attention should be paid to such lesions with uncharacteristic radiographic appearances to avoid misdiagnosis.

## References

[1] Rahimi A Beabout JW Ivins JC Chondromyxoid fibroma: a clinicopathologic study of 76 cases. Cancer 1972;30:726–36. 5075357 10.1002/1097-0142(197209)30:3<726::aid-cncr2820300321>3.0.co;2-t

[2] Jaffe HL Lichtenstein L Chondromyxoid fibroma of bone: a distinctive benign tumor likely to be mistaken especially for chondrosarcoma. Arch Pathol 1948;45:541–51. 18891025

[3] Hakan T Vardar Aker F Chondromyxoid fibroma of frontal bone: a case report and review of the literature. Turk Neurosurg 2008;18:249–53. 18814113

[4] Karkuzhali P Chitraklekha S Muthuvel E Chondromyxoid fibroma of the parietal bone. Neuropathology 2005;25:84–8. 15822822 10.1111/j.1440-1789.2004.00576.x

[5] Feuvret L Noel G Calugaru V Chondromyxoid fibroma of the skull base: differential diagnosis and radiotherapy: two case reports and a review of the literature. Acta Oncol 2005;44:545–53. 16165913 10.1080/00365590500237846

[6] Haberal AN Bilezikci B Coskun M Unusual presentation of a chondromyxoid fibroma of the temporal bone. Tr J of Med Sci 2001;31:91–3.

[7] Xu H Qin Z Shi Z Chondromyxoid fibroma in the sella turcica region. J Clin Neurosci 2011;18:1419–21. 21778057 10.1016/j.jocn.2011.01.035

[8] Ostrowski ML Spjut HJ Bridge JA Chondromyxoid fibroma. In: Fletcher CDM, Unni KK, Mertens F, eds. World Health Organization Classification of Tumours. Pathology and Genetics of Tumours of Soft Tissue and Bone. Lyon: IARC Press; 2002:243.

[9] Morris LG Rihani J Lebowitz RA Chondromyxoid fibroma of sphenoid sinus with unusual calcifications: case report with literature review. Head Neck Pathol 2009;3:169–73. 19644549 10.1007/s12105-009-0121-6 PMC2715466

[10] Bloom KK Ellen J Kaye D Occipital neuralgia and twelfth nerve palsy from a chondromyxoid fibroma. J Ky Med Assoc 2004;102:255–8. 15216723

[11] Morimura T Nakano A Matsumoto T Chondromyxoid fibroma of the frontal bone. AJNR Am J Neuroradiol 1992;13:1261–4. 1636548 PMC8333569

[12] Wu CT Inwards CY O’Laughlin S Chondromyxoid fibroma of bone: a clinicopathologic review of 278 cases. Hum Path 1998;29:438–46. 9596266 10.1016/s0046-8177(98)90058-2

[13] Ezzat S Asa SL Couldwell WT The prevalence of pituitary adenomas: a systematic review. Cancer 2004;101:613–9. 15274075 10.1002/cncr.20412

[14] Asa Sylvia L Practical pituitary pathology: what does the pathologist need to know? Arch Pathol Lab Med 2008;132:1231–40. 18684022 10.5858/2008-132-1231-PPPWDT

[15] Curran JG O’Connor E Imaging of craniopharyngioma. Childs Nerv Syst 2005;21:635–9. 16078078 10.1007/s00381-005-1245-y

[16] Cugati G Singh M Symss NP Primary intrasellar schwannoma. J Clin Neurosci 2012;19:1584–5. 22959445 10.1016/j.jocn.2011.09.041

[17] Bonneville JF Bonneville F Cattin F Magnetic resonance imaging of pituitary macroadenomas. Eur Radiol 2005;15:543–8. 15627195 10.1007/s00330-004-2531-x

[18] Hamid R Sarkar S Hossain MA Clinical picture of craniopharyngioma in childhood. Mymensingh Med J 2007;16:123–6. 17703145

[19] Garrè ML Cama A Craniopharyngioma: modern concepts in pathogenesis and treatment. Curr Opin Pediatr 2007;19:471–9. 17630614 10.1097/MOP.0b013e3282495a22

[20] Ahmet A Blaser S Stephens D Weight gain in craniopharyngioma-a model for hypothalamic obesity. J Pediatr Endocrinol Metab 2006;19:121–7. 16562584 10.1515/jpem.2006.19.2.121

[21] Sartoretti-Schefer S Wichmann W Aguzzi A MR differentiation of adamantinous and squamous-papillary craniopharyngiomas. AJNR Am J Neuroradiol 1997;18:77–87. 9010523 PMC8337875

[22] Eldevik OP Blaivas M Gabrielsen TO Craniopharyngioma: radiologic and histologic findings and recurrence. AJNR Am J Neuroradiol 1996;17:1427–39. 8883637 PMC8338733

[23] Skolnik AD Loevner LA Sampathu DM Cranial nerve schwannomas: diagnostic imaging approach. Radiographics 2016;36:1463–77. 27541436 10.1148/rg.2016150199

[24] Louis DN Ohgaki H Wiestler OD The 2007 WHO classification of tumors of the central nervous system. Acta Neuropathol 2007;114:97–109. 17618441 10.1007/s00401-007-0243-4 PMC1929165

